# Flea index predicts plague epizootics among great gerbils (*Rhombomys opimus*) in the Junggar Basin China plague focus

**DOI:** 10.1186/s13071-022-05330-7

**Published:** 2022-06-17

**Authors:** Wenting Mou, Bo Li, Xiaojun Wang, Ying Wang, Peihua Liao, Xiaobing Zhang, Youjun Gui, Guliayi Baokaixi, Yongjun Luo, Mukedaisi Aihemaijiang, Qiguo Wang, Feng Liu

**Affiliations:** 1 Microbiological Laboratory, Urumqi Center for Disease Control and Prevention, Urumqi, China; 2 Department of Emergency Response and Plague Control, Xinjiang Center for Disease Control and Prevention, Urumqi, China; 3 Department of Human Resource, Xinjiang Center for Disease Control and Prevention, Urumqi, China; 4 Department of Science and Education, Xinjiang Center for Disease Control and Prevention, Urumqi, China

**Keywords:** Great gerbils, Flea index, Plague epizootic, Junggar Basin plague focus, Prediction

## Abstract

**Background:**

The Junggar Basin plague focus was the most recently identified natural plague focus in China. Through extensive field investigations, great gerbils (*Rhombomys opimus*) have been confirmed as the main host in this focus, and the community structure of their parasitic fleas is associated with the intensity of plague epizootics. The aim of this study is to provide an indicator that can be surveyed to evaluate the risk of plague epizootics.

**Methods:**

Between 2005 and 2016, rodents and fleas were collected in the Junggar Basin plague focus. The parasitic fleas on great gerbils were harvested, and anti-F1 antibody in the serum or heart infusion of great gerbils was detected through indirect hemagglutination assay. *Yersinia pestis* (*Y. pestis*) was isolated from the liver and spleen of great gerbils and their parasitic fleas using Luria-Bertani plates. Receiver-operating characteristic (ROC) curve was used to evaluate the predictive value of flea index.

**Results:**

Between 2005 and 2016, 98 investigations were performed, and 6778 great gerbils and 68,498 fleas were collected. Twenty-seven rodents were positive for *Y. pestis* isolation with a positivity rate of 0.4%; 674 rodents were positive for anti-F1 antibody with a positivity rate of 9.9%. Among these 98 investigations, plague epizootics were confirmed in 13 instances by *Y. pestis*-positive rodents and in 59 instances by anti-F1 antibody-positive rodents. We observed a higher flea index among rodents with confirmed plague epizootic compared to the negative ones (*P* = 0.001, 0.002), with an AUC value of 0.659 (95% *CI*: 0.524–0.835, *P* = 0.038) for *Y. pestis*-positive rodents and an AUC value of 0.718 (95% *CI*: 0.687–0.784, *P* < 0.001) for anti-F1 antibody-positive rodents.

**Conclusions:**

Significantly higher flea index was associated with confirmed plague epizootic cases among great gerbils and could be used to predict plague epizootics in this focus.

**Graphical Abstract:**

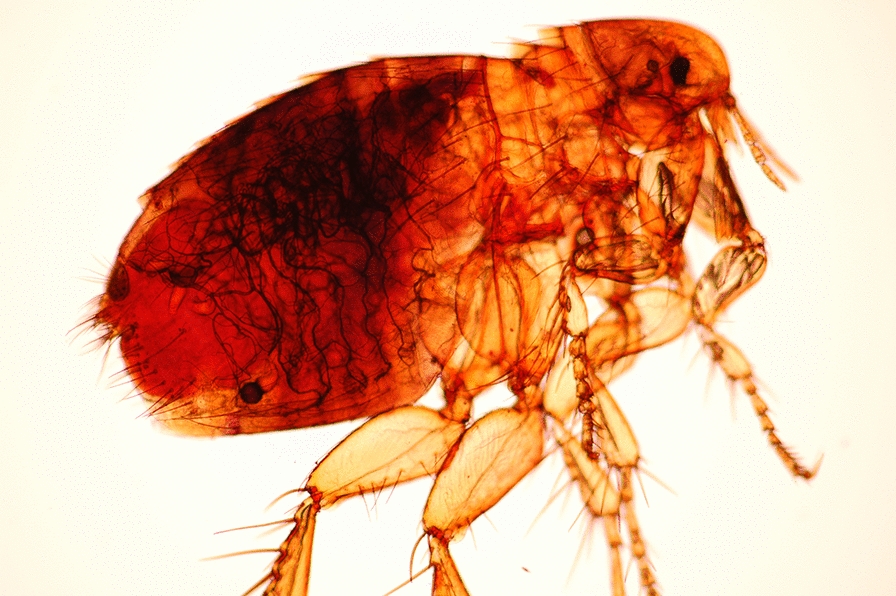

## Background

Plague, caused by infection of *Yersinia pestis*, is a re-emerging zoonotic disease characterized by its ability to persist in certain natural systems, i.e. plague foci [1, 2]. In plague foci, the pathogen, hosts and vectors interact with each other, enabling plague to be maintained in the foci for a long period of time. Plague has a complicated ecology characterized by different degrees of vectorial capacity among different flea species and different degrees of susceptibility among hosts.

So far, 15 natural plague foci have been identified in China, covering > 1.4 million km^2^ of land [3, 4]. Among them, the Junggar Basin plague focus was most recently identified in 2005 [5]. Between 2005 and 2016, extensive field investigations were performed to understand the distribution, fauna and population structure of host animals and their parasitic fleas as well as the dynamic of plague epizootics of this focus. The results showed that 84.4% of *Y. pestis* strains isolated in this focus were from great gerbils (*Rhombomys opimus*) and their parasitic fleas. Great gerbils, widely distributed in clay or sandy desert areas throughout Central Asia, have been confirmed as the main host in this focus [6, 7], and the community structure of their parasitic fleas is associated with the intensity of plague epizootics [8]. As an infection source for humans in this focus, great gerbils infected by *Y. pestis* can transmit plague to humans through direct contact or their fleas. Therefore, zoonotic plague surveillance is critical for preventing the spread of animal plague to humans.

Studies demonstrate that the abundance of the fleas on hosts is an important indicator for plague control in many plague foci [9–13]. Moreover, the index of specific fleas can be applied in the prediction of plague activity among hut-dwelling rats [14]. However, the association of the flea index with plague epizootics among great gerbils in the Junggar Basin plague focus and its predictive value have still not been analyzed. The zoonotic plague surveillance in this focus between 2005 and 2016, collecting data on etiological and serological testing of rodents, etiological testing of parasitic fleas on rodents, flea indexes, etc., provided us the opportunity to evaluate the potential of the flea index on great gerbils for predicting plague epizootics among great gerbils. The aim of this analysis is to provide an indicator that can be surveyed to evaluate the risk of plague epizootics.

## Methods

### Animals

Between 2005 and 2016, the zoonotic plague surveillance aiming for rodents was performed in 15 counties of the Junggar Basin plague focus during the plague season (April, May, September and October) according to the methods described by Dennis et al. [15]. These 15 counties were Manasi, Karamay, Hefeng, Alashankou, Wusu, Jinghe, Shawan, Mulei, Qitai, Jimsar, Fukang, Midong, Buerjin, Changji and Hutubi. Traps for great gerbils were set from 10 a.m. to 18 p.m. and inspected every half hour. Trapped rodents were immediately put in white cloth bags after collecting cardiac blood to prevent flea dissociation from their bodies. Traps for nocturnal rodents were set from late afternoon (20 p.m.) to the next morning (8 a.m.) to prevent flea dissociation from their bodies. Traps were set for at least 2 consecutive days in each observation.

### Fleas

Fleas were collected from captured rodents after they were anaesthetized using diethyl ether and placed in a white basin. Fleas were then removed by brushing captured rodents with a tooth brush and collected into small vials with ophthalmic forceps. Fleas were identified by flea morphologists using light microscopy. The flea index was calculated by dividing the number of fleas collected from great gerbils by the total number of great gerbils, i.e. number of fleas per individual rodents [15].

### Laboratory detection

The anti-F1 antibody in the serum or heart infusion of great gerbils was detected through indirect hemagglutination assay (IHA), and *Y. pestis* was isolated from the liver and spleen of great gerbils using Luria-Bertani (LB) plates at 28 °C [6]. Plague epizootics were confirmed by anti-F1 antibody- or *Y. pestis*-positive rodents. The conspecific fleas on the conspecific rodents captured in the same observation were divided into different pools (about 30 each pool). The fleas were then ground with normal saline, and the suspension was used for *Y. pestis* isolation [5].

### Statistical analysis

Statistical analysis was conducted with SPSS version 17.0 (SPSS Inc., USA), and significance was set at two-sided *P* < 0.05. The distribution of the flea index of great gerbils was evaluated using Kolmogorov-Smirnov test, and the flea index was described using mean ± standard deviation and compared using Student's *t* test. Receiver-operating characteristic (ROC) curve was used to evaluate the predictive value of the flea index for plague epizootics. In the ROC analysis, the status variable was set as whether plague epizootics were confirmed in these 15 counties according to the results of each investigation, and the corresponding flea index was set as the test variable. Area under curve (AUC) was compared using *Z* test. Sensitivity, specificity and accuracy were calculated.

## Results

### Surveillance data

Between 2005 and 2016, a total of 98 investigations were performed in the 15 counties of the Junggar Basin plague focus. Fourteen species of 11,760 rodents were captured, mainly including *Rhombomys opimus* (great gerbils), *Meriones meridianus* (*M. meridianus*), *M. erythrourus*, *M. tamariscinus*, *Dipus sagitta* etc., and 19 species of 72,883 parasitic fleas were collected, mainly including *Xenopsylla minax* (*X. minax*), *X. skrjabini*, *X. hirtipes*, *X. conformis*, *Echidnophaga oschanini*, etc. Among them, great gerbils accounted for 57.6% (6778/11760), and their parasitic fleas accounted for 94.0% (68498/72883). Twenty-seven great gerbils were positive for *Y. pestis* isolation with a positivity rate of 0.4%. All the 68,498 parasitic fleas were divided into 2186 pools for *Y. pestis* isolation, and 12 pools were positive for *Y. pestis* isolation with a positivity rate of 0.5%; 674 great gerbils were positive for anti-F1 antibody with a positivity rate of 9.9%. The rate of *Y. pestis*-positive great gerbils was much lower than that of anti-F1 antibody-positive great gerbils (0.4% vs. 9.9%, *χ*^*2*^ = 629.724, *P* < 0.001). Among these 98 investigations, the occurrence of plague epizootics was confirmed in 13 cases by the presence of *Y. pestis*-positive great gerbils and in 59 cases by the presence of anti-F1 antibody-positive great gerbils.

### Flea index of great gerbils in positive and negative observations

**Fig. 1:**
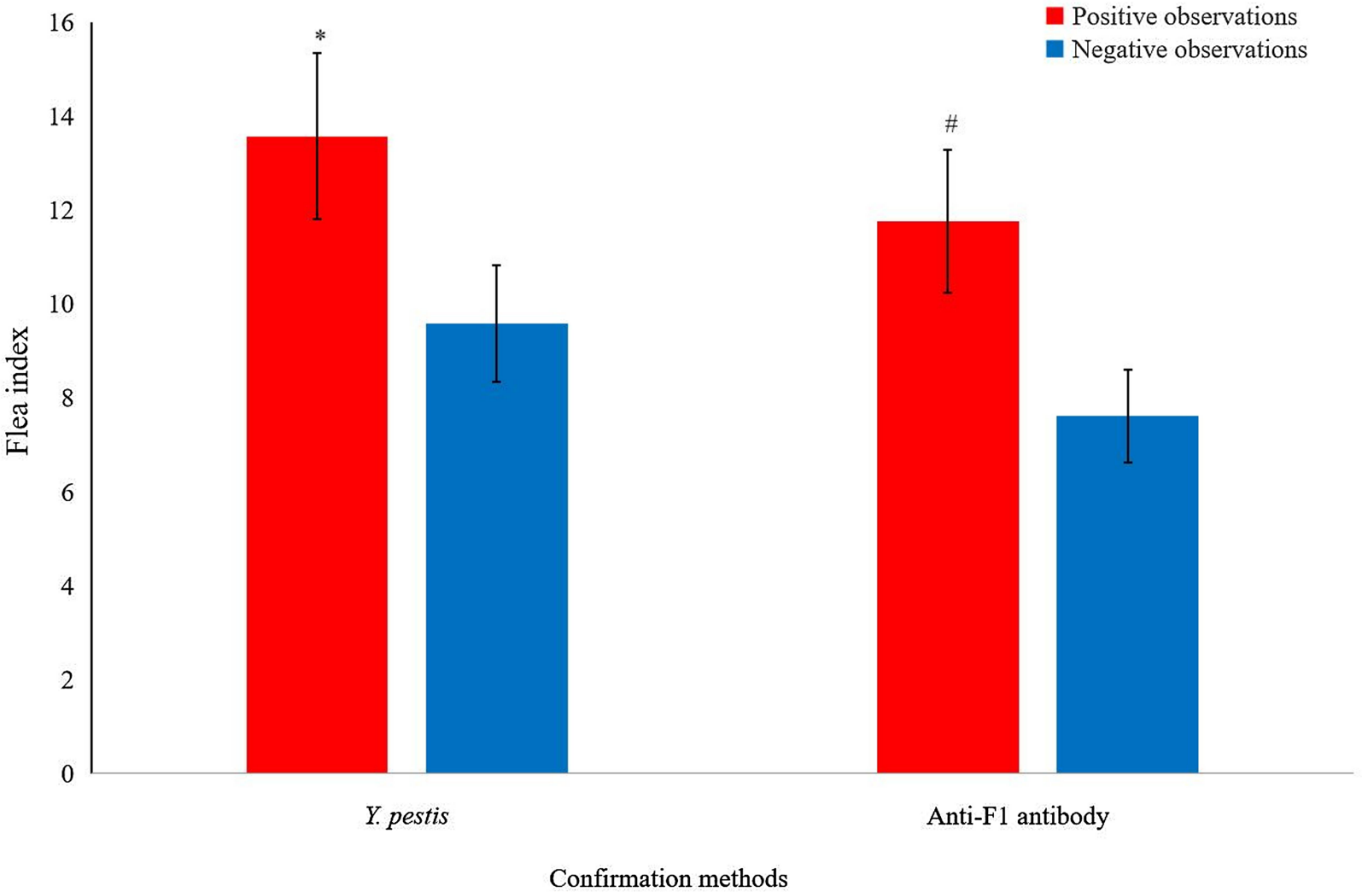
Flea index of great gerbils in positive/negative observations confirmed by *Y. pestis* culture with LB plates and confirmed by anti-F1 positive/negative for the immunoassay. *t_(96)_ = 3.379, *P* = 0.001, ^#^: *t*_(96)_ = 3.127, *P* = 0.002; both represented significant differences

In this study, positive observations (plague epizootics) were characterized by the occurrence of *Y. pestis*-positive rodents or anti-F1 antibody-positive rodents and negative observations (situations); *Y. pestis*-negative and anti-F1 antibody-negative rodents were detected. The flea index of all 6778 great gerbils was 10.11. Student's *t*-test showed that the flea index of great gerbils in positive observations confirmed by *Y. pestis*-positive rodents (13 cases) was higher than that in negative observations, and the flea index of great gerbils in positive observations confirmed by anti-F1 antibody-positive rodents (59 cases) was also higher than that in negative observations (Fig. 1).

### Predictive value of the flea index for plague epizootics

**Fig. 2:**
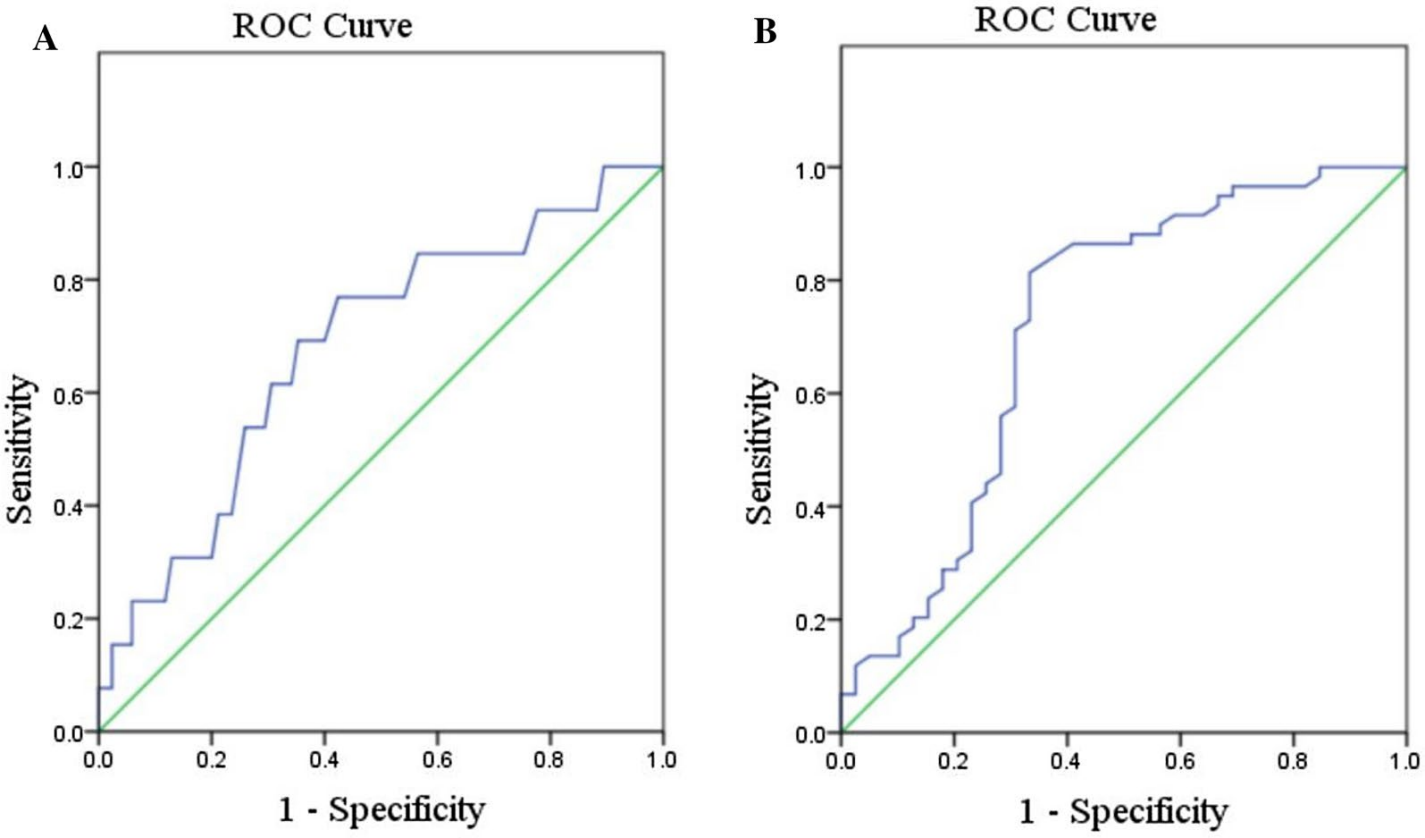
**A** ROC curve of the flea index for predicting plague epizootics confirmed by *Y. pestis*-positive great gerbils; **B** ROC curve of the flea index for predicting plague epizootics confirmed by anti-F1 antibody-positive great gerbils

The ROC curve (Fig. 2A) showed that the AUC of the flea index was 0.659 (*SE*: 0.077, 95% *CI*: 0.524–0.835, *P* = 0.038) when it was used to predict plague epizootics confirmed by presence of *Y. pestis*-positive great gerbils. Its best cutoff was 11.84, and the prediction results are shown in Table 1A. The sensitivity, specificity and accuracy were 69.2%, 56.5% and 58.2%, respectively.

**Table 1:** Prediction results of the flea index for plague epizootics confirmed by *Y. pestis*-positive great gerbils **A** and anti-F1 antibody-positive great gerbils (**B**)

Prediction criterion		Plague epizootic		Total
		Yes	No	
A	Flea index ≥ 12.63 (positive)	9	37	46
	Flea index < 12.63 (negative)	4	48	52
	Total	13	85	98
B	Flea index ≥ 10.59 (positive)	47	14	61
	Flea index < 10.59 (negative)	12	25	37
	Total	59	39	98

The ROC curve (Fig. 2B) showed that the AUC of the flea index was 0.718 (*SE*: 0.057, 95% *CI*: 0.687–0.784, *P* < 0.001) when it was used to predict plague epizootics confirmed by presence of anti-F1 antibody-positive great gerbils. Its best cutoff was 10.59, and the prediction results are also shown in Table 1B. The sensitivity, specificity and accuracy were 79.7%, 64.1% and 73.5%, respectively. The AUC of the flea index for the prediction of plague epizootics confirmed by presence of anti-F1 antibody-positive rodents was not statistically different from that for the prediction of plague epizootics confirmed by presence of *Y. pestis*-positive rodents (*Z* = 0.616, *P* = 0.546).

## Discussion

Many studies have already explored the feasibility of the flea index as an evaluation tool for epizootics and human plague [14, 16–22], but it remains controversial. Eisen et al. [14] monitored the *Xenopsylla* flea index in hut-dwelling rats in sentinel villages in the West Nile region of Uganda and evaluated its predictive value for plague occurrence. Their results showed that the *Xenopsylla* flea index increased preceding the start of the annual plague season and tended to be greater in the years when plague activity was reported than in the years when plague activity was not. *Xenopsylla* flea index > 1 yielded a sensitivity of 46.7% and positive predictive value (PPV) of 22.6% for the prediction of plague activity. When the flea index decreased to 0.50 or 0.75, the sensitivity increased to 60.0% and 53.3%, respectively, and the PPV decreased to 12.5% and 17.4%, respectively. Laudisoit et al. [21] demonstrated that *Pulex irritans* was the predominant flea species in houses of Lushoto District, Tanzania, and its density was associated with the logarithmically transformed plague incidence and plague frequency. However, Brinkerhoff et al. [22] investigated whether increased flea abundance predisposed blacked-tailed prairie dogs to infection by *Y. pestis* by tracking flea occurrence for 3 years at prairie dog colony sites before, during and after a local plague epizootic. Their results showed that there were no significant differences in preepidemic flea prevalence and abundance between plague-negative and -positive colonies. Moreover, there were no significant differences in flea prevalence and abundance between before and after plague at either plague-negative or -positive sites. Therefore, they concluded that flea abundance could not be used to predict plague epizootics among black-tailed prairie dogs in Boulder County, Colorado, USA.

The ROC analysis can detect the predictive power of the flea index at arbitrary thresholds for plague epizootics and derive the best cutoff. Moreover, the predictive values can be compared using AUCs by *Z* test. Therefore, we used the ROC analysis instead of other statistical methods. Our results demonstrated that the flea index of great gerbils in the positive observations in which plague epizootic was confirmed was higher than that in the negative observations where plague epizootics were not. The flea index yielded an AUC of 0.659 for the prediction of plague epizootic confirmed by presence of *Y. pestis*-positive great gerbils and an AUC of 0.718 for the prediction of plague epizootic confirmed by presence of anti-F1 antibody-positive great gerbils. The AUC of the flea index for the prediction of plague epizootics confirmed by presence of anti-F1 antibody-positive rodents was slightly elevated compared with that for the prediction of plague epizootics confirmed by presence of *Y. pestis*-positive rodents, but the difference was not statistically significant. The reason may be that positive anti-F1 antibody represents a previous epizootic and isolation of *Y. pestis* represents an on-going epizootic. The flea index yielded a sensitivity of 79.7% and specificity of 64.1% for the prediction of plague epizootic confirmed by presence of anti-F1 antibody-positive rodents and yielded a sensitivity of 69.2% and specificity of 56.5% for the prediction of plague epizootic confirmed by presence of *Y. pestis*-positive rodents.

The community structure of parasitic fleas on great gerbils was complicated, demonstrating an average richness of 1.66 and an average diversity of 1.5556 [8]. The flea index of great gerbils was much higher than that of other hosts, for example small mammals in the West Nile region of Uganda and black-tailed prairie dogs [14, 22]. In addition, the carrier rate of *Y. pestis* in the predominant flea species of great gerbils was high, and 12 strains of *Y. pestis* were isolated during the same period (2005–2016). These may partly explain the relatively high predictive value of the flea index for plague epizootics among great gerbils in the Junggar Basin plague focus. Isolation of *Y. pestis* is a key indicator to determine the prevalence of plague epizootics. However, the detection rate of *Y. pestis* is affected by many factors such as workload, experimental conditions and operation skills, which is confirmed by our results. In this study, the rate of *Y. pestis*-positive great gerbils was much lower than that of anti-F1 antibody-positive great gerbils (0.4% vs. 9.9%, *χ*^*2*^ = 629.724, *P* < 0.001). Moreover, the 13 cases confirming the occurrence of plague epizootics by the presence of *Y. pestis*-positive rodents were included in the 59 cases confirmed by the presence of anti-F1 antibody-positive rodents. Therefore, positive anti-F1 antibody was also used as the criterion for the occurrence of plague epizootics in this study.

## Conclusions

More than a decade of data collected from the Junggar Basin plague focus supported that significantly higher flea index was associated with confirmed plague epizootics cases among great gerbils. Our results demonstrated that the flea index was an accurate parameter that could be used to predict plague epizootics in this focus.

## Data Availability

The datasets used and/or analyzed during the current study are available from the corresponding author on reasonable request.

## Abbreviations

*Y. pestis*: *Yersinia pestis*
IHA: Indirect hemagglutination assay
LB: Luria-Bertani
ROC: Receiver-operating characteristic
AUC: Area under curve
*SE*: Standard error
*CI*: Confidence interval

## References

[1] Bertherat E (2019). Plague around the world in 2019. Weekly Epidemiol Rec.

[2] Vallès X, Stenseth NC, Demeure C, Horby P, Mead PS, Cabanillas O (2020). Human plague: an old scourge that needs new answers. PLoS Negl Trop Dis.

[3] Li Y, Cui Y, Hauck Y, Platonov ME, Dai E, Song Y (2009). Genotyping and phylogenetic analysis of Yersinia pestis by MLVA: insights into the worldwide expansion of Central Asia plague foci. PLoS ONE.

[4] Li Y, Dai E, Cui Y, Li M, Zhang Y, Wu M (2008). Different region analysis for genotyping Yersinia pestis isolates from China. PLoS ONE.

[5] Zhang YJ, Dai X, Jiang W, Wang XH, Li B, Lei G (2008). Study on the situation of plague in Junggar Basin of China. Zhonghua Liu Xing Bing Xue Za Zhi.

[6] Zhang Y, Dai X, Wang X, Maituohuti A, Cui Y, Rehemu A (2012). Dynamics of Yersinia pestis and its antibody response in great gerbils (*Rhombomys opimus*) by subcutaneous infection. PLoS ONE.

[7] Zhang Y, Luo T, Yang C, Yue X, Guo R, Wang X (2018). Phenotypic and molecular genetic characteristics of Yersinia pestis at an emerging natural plague focus, Junggar Basin. China Am J Trop Med Hyg.

[8] Zhang YJ, Luo T, Abu L, Wang QG, Abu L (2013). Community structure of great gerbils (*Rhombomys opimus*) parasitic fleas in Junggar Basin focus and related epizootiological characteristics on plague. Zhonghua Liu Xing Bing Xue Za Zhi.

[9] Yin JX, Dong XQ, Liang Y, Wang P, Siriarayaporn P, Thaikruea L (2007). Human plague outbreak in two villages, Yunnan Province, China, 2005. Southeast Asian J Trop Med Public Health.

[10] Wilder AP, Eisen RJ, Bearden SW, Montenieri JA, Tripp DW, Brinkerhoff RJ (2008). Transmission efficiency of two flea species (Oropsylla tuberculata cynomuris and Oropsylla hirsuta) involved in plague epizootics among prairie dogs. EcoHealth.

[11] Tripp DW, Gage KL, Montenieri JA, Antolin MF (2009). Flea abundance on black-tailed prairie dogs (*Cynomys ludovicianus*) increases during plague epizootics. Vector Borne Zoonotic Dis.

[12] Salkeld DJ, Salathé M, Stapp P, Jones JH (2010). Plague outbreaks in prairie dog populations explained by percolation thresholds of alternate host abundance. Proc Natl Acad Sci U S A.

[13] Yin JX, Geater A, Chongsuvivatwong V, Dong XQ, Du CH, Zhong YH (2011). Predictors for abundance of host flea and floor flea in households of villages with endemic commensal rodent plague, Yunnan Province, China. PLoS Negl Trop Dis.

[14] Eisen RJ, Atiku LA, Mpanga JT, Enscore RE, Acayo S, Kaggwa J (2020). An evaluation of the flea index as a predictor of plague epizootics in the west Nile region of Uganda. J Med Entomol.

[15] Dennis DT, Gage KL, Gratz N, Poland JD, Tikhomirov E. Plague manual: epidemiology, distribution, surveillance and control. WHO/CDS/CSR/EDC/992.Accessed date: 6 April 2022. https://apps.who.int/iris/handle/10665/66010.

[16] Olson WP (1969). Rat-flea indices, rainfall, and plague outbreaks in Vietnam, with emphasis on the Pleiku area. Am J Trop Med Hyg.

[17] Njunwa KJ, Mwaiko GL, Kilonzo BS, Mhina JI (1989). Seasonal patterns of rodents, fleas and plague status in the Western Usambara Mountains. Tanzania Med Vet Entomol.

[18] Chanteau S, Ratsifasoamanana L, Rasoamanana B, Rahalison L, Randriambelosoa J, Roux J (1998). Plague, a reemerging disease in Madagascar. Emerg Infect Dis.

[19] Pham HV, Dang DT, Tran Minh NN, Nguyen ND, Nguyen TV (2009). Correlates of environmental factors and human plague: an ecological study in Vietnam. Int J Epidemiol.

[20] Jones SD, Atshabar B, Schmid BV, Zuk M, Amramina A, Stenseth NC (2019). Living with plague: lessons from the soviet union's antiplague system. Proc Natl Acad Sci U S A.

[21] Laudisoit A, Leirs H, Makundi RH, Van Dongen S, Davis S, Neerinckx S (2007). Plague and the human flea, Tanzania. Emerg Infect Dis.

[22] Brinkerhoff RJ, Collinge SK, Ray C, Gage KL (2010). Rodent and flea abundance fail to predict a plague epizootic in black-tailed prairie dogs. Vector Borne Zoonotic Dis.

